# Case report: Generalized bullous fixed drug eruption mimicking epidermal necrolysis

**DOI:** 10.3389/fmed.2023.1125754

**Published:** 2023-08-14

**Authors:** Maren Paulmann, Felix Reinkemeier, Marcus Lehnhardt, Maja Mockenhaupt

**Affiliations:** ^1^Dokumentationszentrum schwerer Hautreaktionen (dZh), Department of Dermatology, Medical Center—University of Freiburg, Freiburg, Germany; ^2^Department of Plastic Surgery and Hand Surgery, Burn Center, Sarcoma Center, Berufsgenossenschaft University Hospital Bergmannsheil Bochum, Bochum, Germany

**Keywords:** case report, generalized bullous fixed drug eruption, epidermal necrolysis, recurrence, re-exposure, metamizole, GBFDE

## Abstract

Generalized bullous fixed drug eruption (GBFDE) is the most severe form of fixed drug eruption and can be misdiagnosed as epidermal necrolysis (EN). We report the case of a 42-year-old male patient presenting with more than 50% skin detachment without defined areas of exanthema or erythema and a history of one prior event of EN caused by acetaminophen (paracetamol), allopurinol, or amoxicillin 1.5 years ago. The initial diagnosis was GBFDE or EN. The histology of a skin biopsy was unable to distinguish between the two diseases. The course of the disease, the later clinical presentation, and the medical and medication history, however, were in favor of a diagnosis of GBFDE with two potentially culprit drugs: metamizole and ibuprofen. Moxifloxacin, enoxaparin sodium, hydromorphone, and insulin human were administered concomitantly, which makes them suspicious as well. Unfortunately, the patient received an additional dose of metamizole, one of the possible causative drugs, and he developed another bullous reaction within 1 month. This led to the diagnosis of GBFDE due to metamizole. This report highlights the challenges of distinguishing two rare diseases and elucidates the importance of distinct clinical presentation and detailed medication history.

## 1. Introduction

Fixed drug eruption (FDE) is an adverse reaction to multiple drugs and sometimes food (1). FDE is characterized by a limited number of well-demarcated solitary erythematous or violaceous patches of round to oval shape leaving hyperpigmentation after healing. Additionally, blisters or erosions can occur on these patches (2–5). An erosive involvement of oral and/or genital mucous membranes is rarely present and if present, then rather mild (3, 4, 6). Re-exposure to the causative drug leads to a same-site recurrence of these patches, whereas the lesions can increase in size and number (1, 5, 7, 8). In rare cases, the patches occur in generalized distribution with extensive detachment. This type of FDE is called generalized bullous fixed drug eruption (GBFDE) (2, 6). In addition to the variant of clearly demarcated patches, there is a variant of GBFDE with diffuse generalized erythema subsequently showing flaccid blisters (4, 9). Both types of GBFDE can resemble the presentation of epidermal necrolysis (EN), and that is why GBFDE is often misdiagnosed as EN (1, 2, 6, 10). EN is a term, which describes a disease spectrum that includes Stevens–Johnson syndrome (SJS) and toxic epidermal necrolysis (TEN) and their overlap (11–14). SJS and TEN represent a continuum of a rare, severe cutaneous adverse reaction, which is characterized by spots and atypical targets with skin detachment and erosive mucous membranes (6, 13, 15). The epidermal detachment in cases of SJS is < 10% of the body surface area (BSA), in TEN, more than 30% BSA, and in SJS/TEN overlap, 10–30% BSA (15).

We present a case with two events of extensive skin detachment within 1 month and a final diagnosis of GBFDE after the second event.

## 2. Case description

**Figure 1:**
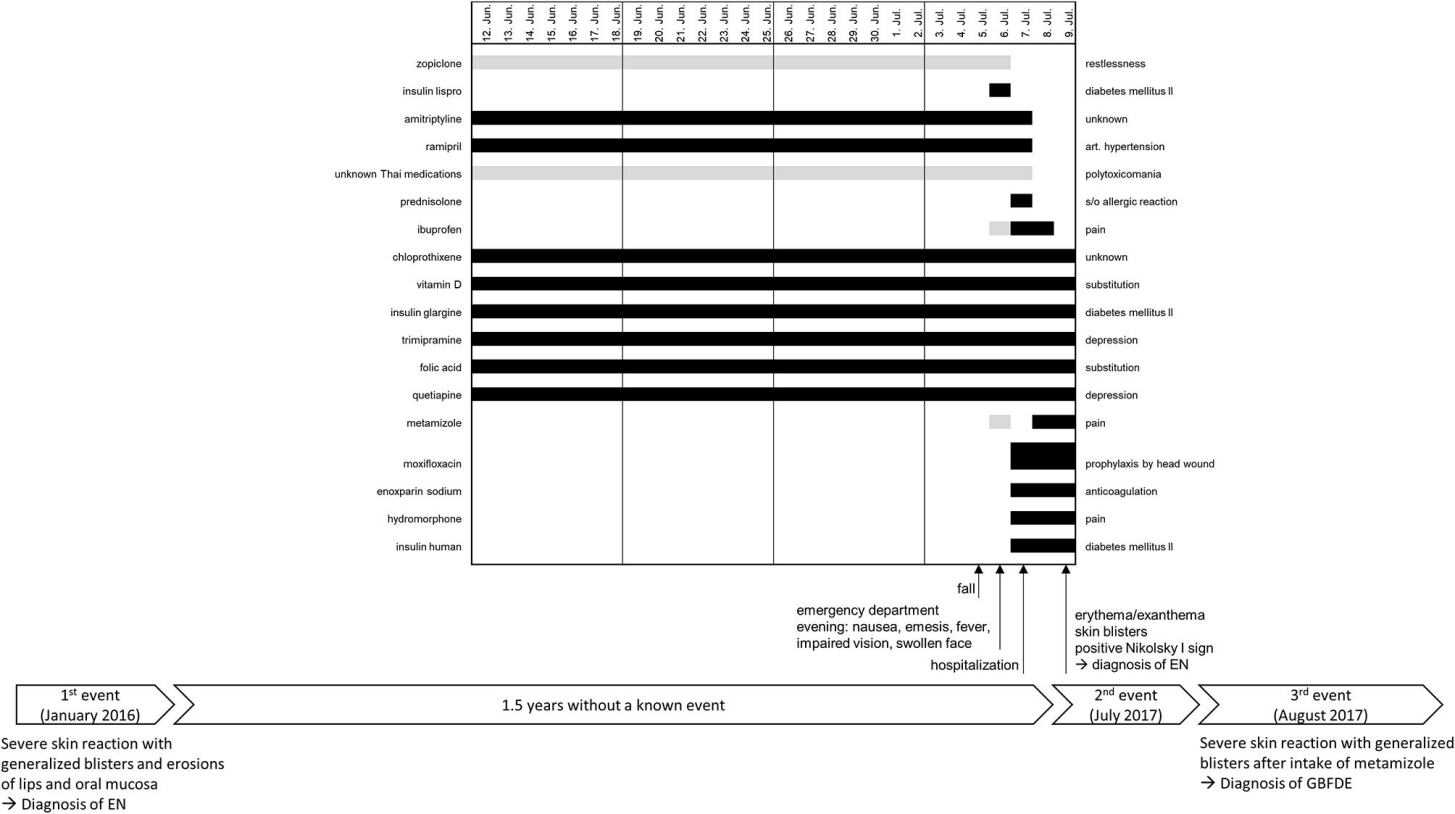
Timeline of the three known events with a detailed medication history of the last 4 weeks before the onset of the severe skin reaction on 9 July 2017. The black bars represent the daily intake of a drug, and the gray bars represent an intermittent or unknown intake. GBFDE, generalized bullous fixed drug eruption; EN, epidermal necrolysis.

A 42-year-old Thai man with a history of polytoxicomania (abuse of alcohol, drugs, and nicotine), arterial hypertension, diabetes mellitus type II, hepatic steatosis, depression, and EN presented to the emergency department due to a fall 1 day before. The head injury required several stitches, and the patient was discharged. In the evening, he experienced deterioration of his general state of health, nausea, emesis, fever, impaired vision, and swelling of his face. Due to further aggravation of these symptoms, the patient presented to the local hospital, where he was admitted to the internal medicine department with hypotension, tachycardia, hypoxia, and increased laboratory values for kidney parameters and C-reactive protein (CRP). Antibiotic treatment with moxifloxacin was initiated, and 100 mg prednisolone i.v. was given. Two days later, he developed a mild exanthema/erythema with skin blisters on the back and limbs as well as a positive Nikolsky I sign, but without mucosal erosions. The consultant dermatologist made a diagnosis of recurrent EN of unknown origin due to taking multiple medications (Figure 1) and took a biopsy. The patient had been treated at this hospital 1 year before because of the acute onset of a severe skin reaction with generalized blisters on the face, back, palms, and soles. In addition, there had been erosions of lips and oral mucosa. The diagnosis by the consultant dermatologist then was EN related to the intake of acetaminophen (paracetamol). allopurinol and amoxicillin were also suspected.

The patient reported that he had not taken acetaminophen (paracetamol) this time but other pain medications (Figure 1). However, there were contradictory statements from the patient and his caretaker regarding 6 July 2017. The patient denied taking metamizole that day, while the caretaker confirmed that he had taken it. The opposite was reported for taking ibuprofen. Nonetheless, both substances were started just before the onset of the reaction (9th July 2017), whereas this short exposure period is only appropriate for GBFDE not for EN. Moxifloxacin, enoxaparin sodium, hydromorphone, and insulin human were also administered during the same period, making them suspicious as well. Considering the relevant period of 4–28 days of drug use to induce EN, no causative drug could be identified. Overall, the drug history should be evaluated with caution due to the polytoxicomania (especially alcohol abuse) of the patient. To determine the cause of the reaction, it was suggested to perform a patch test *in loco* within 2–6 months after discharge.

**Figure 2:**
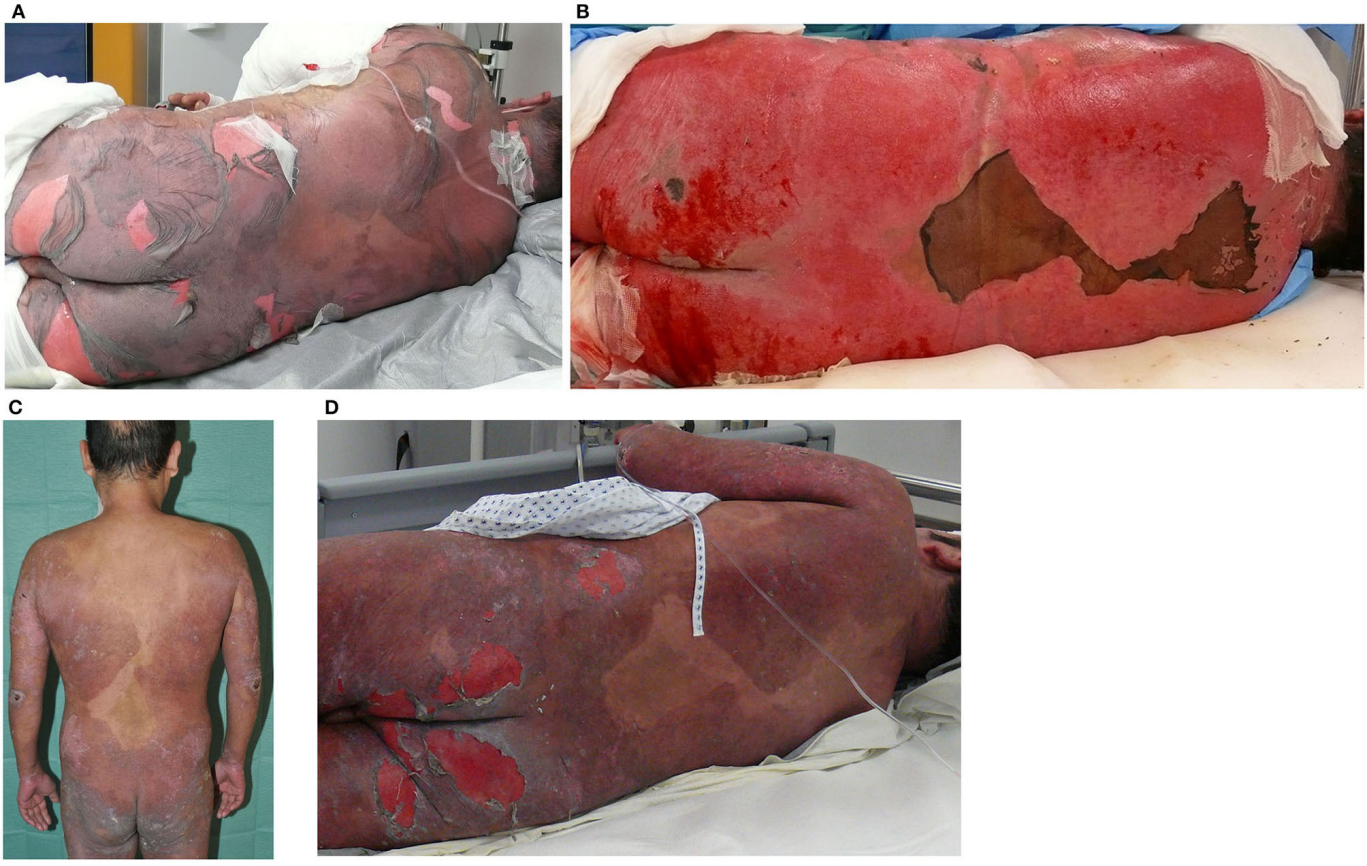
Evolution of the severe skin reaction on the back and buttocks. **(A)** Extensive epidermal detachment: large areas of detachable skin without marked erythema or exanthema (10 July 2017); **(B)** healing phase: almost the entire back and buttocks are detached (19 July 2017), **(C)** the previously affected sites heal, leaving well-demarcated residual hyperpigmentation (31 July 2017), and **(D)** epidermal detachment: areas of detachable skin to a lesser extent (3 August 2017) compared to the reaction 1 month before.

The skin biopsy showed a subepidermal blister and detached epidermis with florid interface dermatitis and many necrotic keratinocytes as well as mild interstitial and perivascular superficial chronic florid inflammation, extravasation of erythrocytes and few eosinophils, edema of the dermis, and discrete pigment incontinence. These findings were compatible with erythema multiforme but could not distinguish between bullous FDE and EN through the histomorphological pattern. For treatment of the severe skin reaction, the patient was placed on non-adhesive wound gauze with fusidic acid on erosive lesions, and systemic therapy with 500 mg prednisolone was started. Despite the initiated therapy, the blisters increased within the next few days resulting in large detached areas of the BSA (more than 30%), and the patient was transferred to a burn unit (Figure 2A). Here, he presented with hypotension, tachycardia, catecholamine requirement, and increased fluid loss due to extensive secreting wounds. After transfer, all previously started drugs as well as long-term medication were discontinued. One day later, erosions of oral mucosa and lips were observed. The treatment was performed with daily changes of dressings with polyhexanide and non-adhesive gauze under short-term anesthesia. Furthermore, systemic treatment with ciclosporin (3 mg per kg body weight) for 10 days was started. Because of the extensive skin detachment, it was not possible at this point to distinguish clinically between EN on large erythema and GBFDE. Within the next few days, the progression stopped and re-epithelialization started (Figure 2B), the patient did no longer require catecholamines, and the topical treatment was modified. During the process of wound healing, the pattern became more suggestive for GBFDE because there were clearly demarcated, large brownish patches (Figure 2C).

After 3 weeks at the burn unit, the patient was transferred back to his local hospital in a cardiopulmonary stable condition and with nearly completed re-epithelialization. After 2 days, the patient experienced generalized pain, for which metamizole 10 drops were accidentally administered. One h later, he developed generalized pruritus, which was treated with dimetindene maleate. Shortly after that, the patient complained about vertigo, fever up to 102.2°F (39°C), hypotension, and tachycardia. He was transferred to the intensive care unit, where he developed skin blisters in a generalized distribution. The symptoms aggravated overnight, and consecutively, the patient was again transferred to the burn unit (Figure 2D). There, he presented with a fever of up to 104.9°F (40.5°C), a catecholamine requirement, but cardiopulmonary stable and with less skin detachment than the month before. Residual post-inflammatory hyperpigmentation could be seen. Supportive care and topical treatment were performed as before but without systemic immunomodulating treatment. Progress of the skin reaction stopped immediately with the detachment of ~10% BSA, the wounds re-epithelialized, and the patient was transferred back to his local hospital after 2 weeks of treatment. After another 4 days, the patient was discharged on 18 August 2017 with a diagnosis of “GBFDE due to metamizole”, which was proven by a second event after accidental re-exposure within a month. Retrospectively, the diagnosis of GBFDE with the detachment of ~55% BSA for the event in July was confirmed through an independent validation process by dermatologists of the RegiSCAR-group (International Registry of Severe Cutaneous Adverse Reactions to drugs and collection of biological samples).

Whether the event 1 year before was also related to metamizole could not be clarified with certainty. At that time, the patient had been drinking heavily on a daily basis, and his memory was thus not reliable.

## 3. Discussion

Fixed drug eruption is a delayed type IV hypersensitivity reaction, which occurs secondary to exposure to a causative agent (5, 16). Each re-exposure to the causative drug leads to lesions that usually recur at previously affected sites (“fixed”). New lesions may also appear on previously not affected skin, whereas old lesions may increase in size (1, 5, 8, 16). Re-exposure to the causative drug after an acute event does not necessarily result in a flare-up of previously involved sites. This is known as the refractory period and can last for weeks or months (17). Generalized bullous fixed drug eruption is a rare and severe variant of FDE with blisters and erosions with involvement of at least 10% of the BSA affecting three of the following six anatomic sites: head/neck, anterior and posterior trunk, upper and lower extremities, and genitalia (18, 19). Due to the generalized distribution with skin detachment, GBFDE can easily be misdiagnosed as EN (1, 18, 20). Even A. Lyell, who introduced the term TEN in 1956, had to acknowledge that two of the four TEN cases in his original report had a diagnosis of GBFDE (12, 21).

Distinguishing GBFDE and EN can be a big challenge as demonstrated by our case with skin detachment of more than 50% BSA. Here, the typical well-demarcated, round or oval erythematous or violaceous patches were not seen at first. Usually, this clinical presentation is a clear distinguishing feature from EN, in which typically a confluent exanthema of macules and/or atypical targets is present (2, 6, 22, 23). When the consensus definition for EN was developed, cases without macules and/or atypical targets but skin detachment of more than 10% BSA were identified. For these cases, the category “TEN without spots” or “TEN on large erythema” was proposed (15). The consensus definition was published in 1993, and experts are now debating whether the few reported cases of “TEN on large erythema” were misclassified and are severe cases of GBFDE (13). The evaluation of a biopsy is often not helpful to distinguish between GBFDE and EN. In both diseases, vacuolar interface dermatitis with necrotic keratinocytes and subepidermal blistering is the most common histopathological pattern (6, 18, 22, 24, 25). Histological features are either individual apoptotic keratinocytes up to clusters in a disseminated distribution or complete epidermal necrosis (6, 26, 27). The dermis reveals a sparse superficial perivascular lymphohistiocytic inflammatory infiltrate (6, 18, 24). In a later stage of GBFDE, a deeper perivascular infiltrate with eosinophils and sometimes neutrophils can be seen, but this pattern is not necessarily indicative (6, 18, 28). The infiltration of eosinophils is more suggestive of GBFDE, and when it is seen, the eosinophils occur in a higher number than in EN (18, 20). Melanophages are also more likely to be associated with GBFDE; in particular, they are present in the late stage and in recurrent events, as they persist in the hyperpigmented areas, revealing pigment incontinence in the histology (18, 29, 30).

Traditionally, it is thought that there is no or rather mild mucosal involvement in GBFDE (1, 22). In a retrospective study, it was shown that in cases of GBFDE, mucosal lesions are more likely present compared with less severe cases of FDE (66.7 vs. 30%) (18). Another study demonstrated that mucosa was involved at one site in 67% of GBFDE cases (2). Compared with EN patients, the mucosal involvement in GBFDE is milder, less pronounced, and often limited to one site, but the mucosa is affected in approximately two-thirds of GBFDE cases (2, 18). In more than 90% of EN cases, involvement of mucous membranes is observed in at least two sites (13). Interestingly, in contrast to EN, ocular mucosa does not seem to be affected in GBFDE. Furthermore, fever and reduced general state are less frequent in GBFDE compared to EN, although GBFDE patients are older (2, 4, 18, 25). It is assumed that GBFDE has a better prognosis than EN (8, 22, 31). However, GBFDE is potentially life-threatening as demonstrated in a large retrospective study matching 58 GBFDE patients with 170 EN patients for age and extent of detachment. The mortality rate did not differ between these two conditions (22% for GBFDE vs. 28% for EN), indicating that especially GBFDE in the elderly deserves the same care and supportive treatment as EN (2, 32).

The two diseases can be distinguished not only based on clinical presentation but also based on medical history (6, 16). GBFDE is a “classical allergic reaction” with sensitization of a susceptible person to a particular drug (or additive or food) with a variable incubation period ranging from a few weeks to many years (5, 27). In addition, sensitization to the particular drug occurs faster with intermittent intake than with continuous use (5). With repeated exposure to the causative drug, the lesions usually occur within 30 min to 48 h (5, 6, 32–34). In contrast, EN patients develop the reaction within the first 8 weeks of treatment, with the majority of the causative drugs being taken in the period of 4–28 days before the onset of EN. In addition, it is the first continuous use of the drug, and there are no previously tolerated exposures in the medication history (6, 35, 36). This leads directly to another distinguishing feature: the presence of previous reactions in cases of GBFDE, which may have been localized and non-bullous. A study from Taiwan found that previous reactions were present in two-thirds of GBFDE cases but were absent in EN cases (18). A GBFDE cohort of 62 patients in Germany also showed that ~62% of the patients had at least one prior event (19). In another study, 38% of the GBFDE patients and 1% of the EN patients reported a previous event (2).

A variety of drugs are associated with FDE. There are geographical differences in the most common causative drugs, sometimes even in the same place over time (1, 26, 29, 37). Anti-infective agents (e.g., ß-lactam antibiotics, tinidazole, and acyclovir), analgesics [e.g., acetaminophen (paracetamol), mefenamic acid, and metamizole], non-steroidal anti-inflammatory drugs (NSAIDs), anti-epileptic drugs (e.g., carbamazepine), psychoactive agents (e.g., barbiturates and codeine), and other miscellaneous drugs (e.g., allopurinol, contrast media, omeprazole, and loratadine) are associated with FDE (7, 18, 26, 38). However, the most common cause of any type of FDE over a long period was the anti-infective drug trimethoprim–sulfamethoxazole, as shown in various studies from different countries (27, 29, 37, 39–42). With decreased use of this sulfonamide combination drug since the 2000s, it has been replaced by naproxen as the most common cause of FDE in Turkey (43). Analgesics are now also identified as the most common cause in many other countries: for example, mefenamic acid in Taiwan (32) and Tunisia (33); etoricoxib, NSAIDs, and acetaminophen (paracetamol) in Singapore (44); and acetaminophen (paracetamol) and NSAIDs in Korea (45). Metamizole is also a known inducer of FDE, with most reports being published before 2000 (37, 46, 47). In recent years, only individual cases of severe GBFDE associated with metamizole were reported (4, 9), which could lead to the impression that the overall number of cases has decreased substantially (29). However, it is striking that a few of the published metamizole-related EN cases more likely seem to be cases of GBFDE, based on the history and the description of the clinical presentation (48–50). The impression that metamizole appears less frequently as a highly suspicious cause can be explained by the fact that it has been withdrawn from the market or never got approved in many countries (e.g., Australia, France, Singapore, and the United States), while in other countries (e.g., Germany, Spain, and Switzerland), it is only available by prescription (51). Nevertheless, in Germany, for example, the number of prescriptions of metamizole almost doubled between 2008 and 2017 leading to an increase of non-allergic and allergic hypersensitivity reactions (52). In many countries (e.g., China, Mexico, Russia, and Turkey), metamizole can be purchased over the counter (51). Due to multiple medications, the causative drug could not be identified in about a quarter of the cases in two studies from Iran and Taiwan (18, 42). Earlier studies have demonstrated that the causative drug could be identified by oral challenge in most cases of GBFDE but not in EN (3, 53). Furthermore, the reaction to the oral re-challenge was completely different between these two diseases. EN could only be provoked in ~10% of the cases by re-challenge with the causative drug but induced discomfort and/or a milder rash. In contrast, GBFDE patients most often reacted to re-challenge with the same pattern (12, 53). Therefore, the oral provocation test is contraindicated in both GBFDE and EN. It is rarely performed even in localized FDE to not trigger GBFDE (1, 54). A patch test is considered safe but less sensitive. Patch testing in FDE or GBFDE is recommended to be performed in previously involved hyperpigmented skin areas if the localization permits. If not, it can be performed as usual on the patient's back. The response rate varies among different studies from 33% to ~80% (18, 33, 55). Therefore, an exact medication history is essential and should also include herbal remedies, over-the-counter medications, and food (6, 38). On the contrary, a patch test is not helpful in EN, since correct positive results are achieved in < 25% of the tests (56). Table 1 summarizes the characteristics of both diseases.

**Table 1:** Characteristics of generalized bullous fixed drug eruption and epidermal necrolysis.

	**Generalized bullous fixed drug eruption**	**Epidermal necrolysis**
Clinical presentation—skin	Well-demarcated, round or oval erythematous or violaceous patches with blisters/erosion on patches OR Diffuse generalized erythema with flaccid blisters	Confluent exanthema of macules and/or atypical targets with blisters/erosions
Clinical presentation—mucosae	1 site in approximately two-thirds of cases (oral, urogenital) Mild and less pronounced	≥2 sites in >90% (oral, ocular, urogenital, and nasal)
Fever and reduced state of health	Less frequent	More frequent
Histological findings	Vacuolar interface dermatitis Necrotic keratinocytes (individual to full-thickness) Sparse to dense inflammatory infiltrate Eosinophils more frequent Melanophages more frequent	Vacuolar interface dermatitis Necrotic keratinocytes (individual to full-thickness) Sparse inflammatory infiltrate Eosinophils rarely present Melanophages rarely present
Latency period (onset after drug intake)	30 min to 48 h	4–28 days
Previously tolerated exposures	Yes, reaction occurs faster with intermittent intake of the culprit drug	No, first continuous use
Previous similar (localized) reaction(s)	Present in up to two-thirds of cases Same-site recurrence with an increase in size and detachment Faster with each recurrence	Present in ≤ 1% No increase in size and detachment Latency stays the same
Testing	Provocation test contraindicated due to the severity of the disease Patch test up to 80% correct positive	Provocation test contraindicated due to the severity of the disease Patch test < 25% correct positive

For treatment, the first step is the identification and removal of the causative drug. Since FDE is a self-limiting disease, supportive therapy is the gold standard and should be adapted according to severity (6, 26, 29).

A case of GBFDE with the detachment of more than 50% BSA is very rare, especially considering that GBFDE itself is a rare and severe variant of FDE and that such a condition can be easily misdiagnosed as EN is reasonable. However, GBFDE and EN are two entities with differences in (1) general condition, (2) clinical presentation, (3) latency period between the beginning of drug use and reaction onset, (4) previous intake, (5) history of previous similar (localized) reaction(s), and (6) pathogenesis. GBFDE is a severe disease that may lead to more extensive skin detachment with each recurrence and deserves the same care and supportive treatment as EN.

## Data availability statement

The original contributions presented in the study are included in the article/supplementary material, further inquiries can be directed to the corresponding author.

## Ethics statement

Written informed consent was obtained from the individual's primary caregiver for the publication of any potentially identifiable images or data included in this article.

## Author contributions

MP drafted the original manuscript. FR, MM, and ML revised the manuscript. All authors approved the final manuscript as submitted for publication.
